# Pediatric Pulmonary Epstein-Barr Virus-Positive Diffuse Large B-Cell Lymphoma: A Case Report and Review of the Literature

**DOI:** 10.1155/2017/8946807

**Published:** 2017-10-08

**Authors:** Eric X. Wei, Roberto F. Silva, James D. Cotelingam, Rodney E. Shackelford

**Affiliations:** Department of Pathology and Translational Pathobiology, LSU Health Shreveport, Shreveport, LA, USA

## Abstract

Non-Hodgkin's lymphoma (NHL) is a common malignancy of childhood; however, a lung primary presentation is an uncommon finding, as is finding an association with the Epstein-Barr virus (EBV). We report the case of a 23-month-old female who developed EBV-associated diffuse large B-cell lymphoma (DLBCL) that was initially thought to be pneumonia. Extensive tissue necrosis, focal angioinvasion, and angiodestruction were observed. She was refractory to various therapy regimens, subsequently developed DLBCL in the central nervous system, and eventually expired. Although EBV+ DLBCL was initially considered to occur predominantly in elderly patients over 50 years of age, it is now increasingly recognized to occur in younger patients with primarily nodal involvement who have overall better prognoses. To our knowledge, this case is the first reported EBV+ DLBCL occurring in a patient below two years of age with lung involvement as the initial clinical presentation.

## 1. Introduction

Lymphomas, including Hodgkin's lymphoma and non-Hodgkin's lymphoma (NHL), constitute the fifth most common malignancy of early childhood, following acute lymphoblastic leukemia/lymphoma, central nervous system tumors, neuroblastoma, and Wilms tumor in a decreasing epidemiologic incidence. Most of the children affected by NHL present with lymphadenopathy or an abdominal or chest mass. The most common mature pediatric NHLs are Burkitt's lymphoma and DLBCL [1–3]. NHLs in childhood are often diagnosed through biopsies after tumor growths are noticed by the parents and infrequently present as pulmonary lesions. Epstein-Barr virus (EBV) is one of the most common viruses in humans and infects more than 90% of the world population. It has transforming cellular capacities capable of promoting B-cell lymphomas [4]. Pediatric EBV+ DLBCL has been reported in developing countries. In Western populations, it is extremely uncommon in immunocompetent young patients.

## 2. Case Representation

A 22-month-old Caucasian female presented to Louisiana State University (LSU) hospital with symptoms of a low-grade fever, cough, decreased activity and oral intake, and an associated bilateral swelling under the jaw line, as reported by her parents. She was born at term with no issues during pregnancy or delivery. She did not have any significant family history of immunodeficiency, although her maternal aunt had lupus, antiphospholipid antibody, autoimmune thyroid, and celiac diseases. She had an incomplete vaccination record and did not receive her 12-month-old vaccinations including Measles, Mumps, Rubella (MMR), Varicella, and Diphtheria, Tetanus, Pertussis (DTaP) #4. She had a history of recurrent otitis media with tympanostomy tube placement and eczema. The patient was tested for Mumps virus due to an incomplete vaccination history and a concern over parotid swelling. Her IgM was positive for the aforementioned virus. Her immunoglobulin levels were all elevated, including IgM, IgG, IgA, and IgE (Immunoglobulin M, G, A, E Flex® Reagent Cartridges). Her IgG subclass levels including those of IgG1, IgG2, IgG3, and IgG4 were all increased. Her respiratory panel for rhinovirus (FilmArray Respiratory Panel) and enterovirus (Cepheid Xpert EV Assay) was positive. Her cytomegalovirus (CMV) (COBAS® AmpliPrep/COBAS TaqMan® CMV Test), human immunodeficiency virus (HIV) (Clearview® COMPLETE HIV 1/2 Assay), and hepatitis panels (COBAS AmpliPrep/COBAS TaqMan HCV Test, v2.0) were negative. The child was leukopenic and was found to have cold agglutinin associated autoimmune hemolytic anemia. On physical exam, there was mild hepatosplenomegaly and mild bilateral cervical lymphadenopathy with 0.5–1 cm mobile lymph nodes. Her EBV viral capsid antigen (VCA) antibodies IgM and IgG were positive at 1.3 and >8.0, respectively; and her early antigen antibody, nuclear antigen antibody, and heterophile antibody were all negative, indicating acute primary infection (BioPlex 2200 EBV IgM and IgG Kits). Her plasma EBV viral loads by quantitative RT-PCR were between 9,000 and 20,600 copy numbers per microliter in serial testing (Viracor Eurofins' Assay, Viracor Laboratories, Lee's Summit, MO). Low levels of CD19+ B-cells (J3-119, Beckman Coulter, Brea, CA), CD3+ (UCHT1, Beckman Coulter), CD4+ (SFCI12T4D11, Beckman Coulter), and CD8+ (SFCI21Thy2D3, Beckman Coulter) T-cells and CD16+ (3G8, Beckman Coulter) and CD56+ (N901, Beckman Coulter) natural killer cells were found by flow cytometric analysis of her peripheral blood. She was given Doxycycline, Vancomycin, and Ceftriaxone. Her blood and urine cultures were negative. During her inpatient hospital stay, her respiratory status deteriorated, which required oxygen infusion and admission to pediatric intensive care unit. Chest X-ray (CXR) and chest computerized tomography (CT) scans found that the patient had bilateral perihilar pulmonary infiltrates with right middle lobe consolidation which were initially interpreted as pneumonia. She was started on Gentamicin and Azithromycin. Her inflammatory markers trended down with her plasma EBV viral load at 9100, and her clinical symptoms improved, although her pulmonary infiltrates persisted per CXR. She was discharged to home temporarily to finish 7 days of Amoxicillin for a total 10-day course of antibiotics for pneumonia. Allergy and immunology service was consulted. She was assessed to possibly have impaired immune function due to her hypergammaglobulinemia and decreased circulating lymphocytes and their subsets. Her EBV load increased to 24,500 a week later and stayed at about 15,000. She was given Vancomycin and Cefepime.

**Figure 1:**
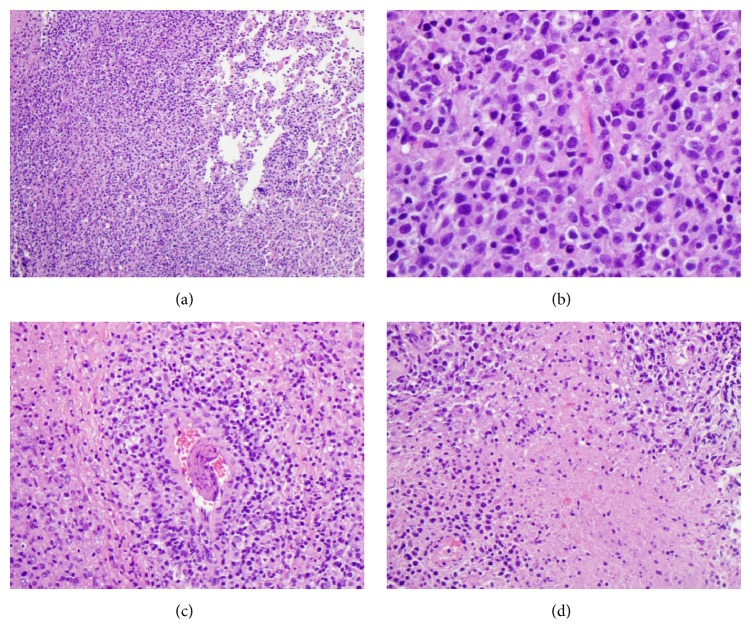
(a) Low-power view shows the tumor cells in a diffuse growth pattern in lung parenchyma and background of uninvolved alveoli (H&E, ×100 magnification). (b) High-power view shows the morphology of the tumor cells in the lung parenchyma (H&E, ×400 magnification). (c) Intermediate power of the tumor cells involving the blood vessel walls with angioinvasion (H&E, ×200 magnification). (d) An intermediate-power view shows the tumor cells with focal necrosis (H&E, ×200 magnification).

**Figure 2:**
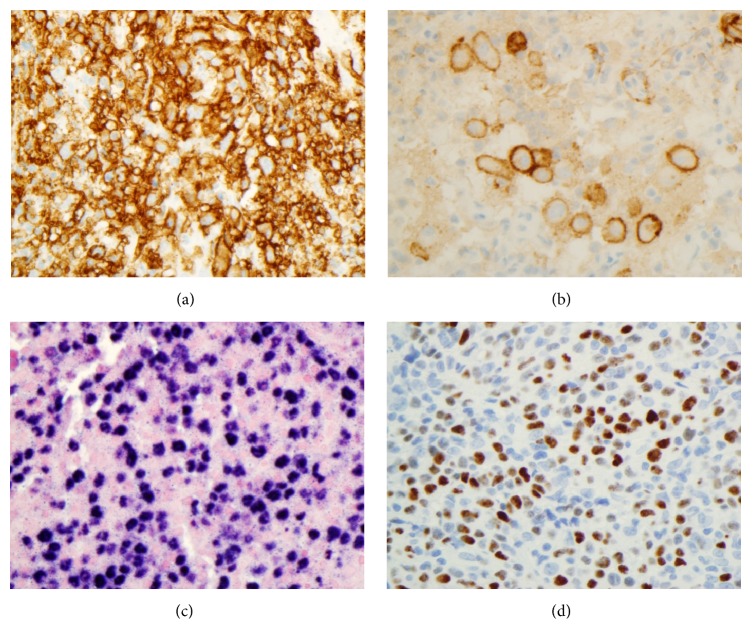
(a) High-power view shows the tumor cells diffusely positive for CD20 (IHC stain with hematoxylin counterstain, ×400 magnification). (b) High-power view shows the tumor cells partially positive for CD10 (IHC stain with hematoxylin counterstain, ×400 magnification). (c) High-power view shows the tumor cells diffusely positive for EBER (ISH stain with eosin counterstain, ×400 magnification). (d) High-power view shows the tumor cells positive for EBNA2 (IHC stain with hematoxylin counterstain, ×400 magnification).

**Figure 3:**
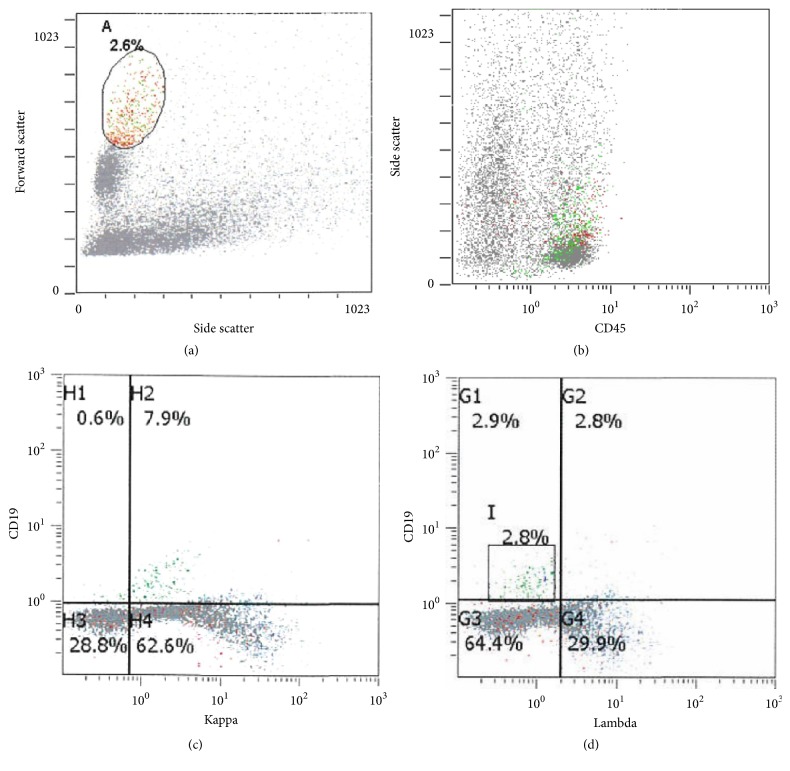
(a) Flow cytometry histogram of forward scatter versus side scatter with a population of large cells gated. (b) Flow cytometry histogram of side scatter versus CD45. (c) Flow cytometry histogram of CD19 versus surface kappa light chain with CD19+ large B-cells highlighted in green events. (d) Flow cytometry histogram of CD19 versus surface lambda light chain with CD19+ large B-cells highlighted in green events.

Due to her febrile neutropenia and associated skin rash, she was readmitted to LSU hospital a month later. CXR showed bilateral pulmonary alveolar and interstitial infiltrates. Her soluble interleukin-2 level was elevated at 3051 U/ml. She did not meet 5 of 8 criteria of hemophagocytic lymphohistiocytosis. A normal neutrophil oxidative burst was observed in the patient's blood sample following phytohaemagglutinin (PHA) stimulation (Dihydrorhodamine Flow Cytometric Test, Mayo Clinic Medical Laboratories, Rochester, MN). Significantly decreased CD45+ (J33, Beckman Coulter) total lymphocyte and CD3+ T-cell proliferative responses were found with PHA stimulation; but normal and robust lymphocyte proliferative response to pokeweed mitogen (PWM) was noted (Invitrogen Click-iT EdU Assay for lymphocyte proliferation to mitogens, Mayo Clinic Medical Laboratories). Three lung biopsy specimens were obtained and all revealed a diffuse proliferation of large atypical cells lacking any significant architectural pattern (Figure 1(a)). Many of these cells had large irregular nuclei with occasional prominent nucleoli (Figure 1(b)). Angioinvasion of the blood vessels was present (Figure 1(c)) along with areas of necrosis (Figure 1(d)). There was a background of small lymphocytes scattered amid the larger more atypical cells. Background alveolar lung parenchyma was present, which was histologically unremarkable. The sheets of large B-cells were CD20-immunopositive (L26, Ventana, Tucson, AZ) (Figure 2(a)). The large atypical lymphoid cells were positive for CD10 (SP67, Ventana) (Figure 2(b)) and MUM1 (MRQ-43, Cell Marque) and negative for BCL-6 (GI191E/A8, Cell Marque) and had a high 90% proliferation index by Ki-67 (30-9, Ventana) immunostain. EBV by in situ hybridization using Epstein-Barr virus-encoded small RNA (EBER) probe (EBER 1 DNP Probe, Ventana) was diffusely positive in about 80% of tumor cells (Figure 2(c)). The lymphoma cells were also positive for CD30 (Ber-H2, Ventana), Epstein-Barr virus latent membrane protein 1 (LMP1) (CS1-4, DAKO, Santa Clara, CA) (performed at Center for Cancer Research, National Institutes of Health, Bethesda, MD), and EBV nuclear antigen 2 (EBNA2) (PE2, Novocastra) (Figure 2(d)), but they were CD15-immunonegative (MMA, Ventana). Special stains for acid-fast bacillus (AFB), AFB-Fite, and Grocott's methenamine silver (GMS) were negative for microorganisms. Flow cytometric analysis of lung biopsy specimens showed a monoclonal large B-cell population with a kappa restriction comprising about 2.8% of total cells (Figures 3(a)–3(d)). Molecular diagnostic studies for immunoglobulin heavy chain frameworks I, II, and III and kappa light chain V-J and V-Kde gene arrangements by PCR were all positive (Invivoscribe Technologies, Inc., San Diego, CA). The FISH assay for MYC gene rearrangement (Abbott Molecular, LSI MYC Dual Color Break Apart Probe) was negative. The patient was subsequently transferred to St. Jude Children's Research Hospital. She was unresponsive to various therapeutic regimens including chemotherapy and cellular therapy (detailed treatment protocols and information were not available for release at the time of manuscript submission) and further developed EBV+ DLBCL lymphoma in the central nervous system and later expired.

## 3. Discussion

DLBCL accounts for 20% of NHLs in the age group of 0 to 14 years [1]. EBV+ DLBCL of the elderly was first recognized as a provisional entity in 2008 in the WHO classification of lymphoid neoplasms and was defined as an EBV+ clonal B-cell lymphoproliferation occurring in patients over 50 years of age without known immunodeficiency or immunosuppression. EBV+ DLBCL of the elderly shows geographic epidemiologic variations and it accounts for 2-3% of DLBCL in Western countries and 8–10% of DLBCL in Asian populations [5]. Recently, EBV+ DLBCL has been increasingly recognized in young immunocompetent patients in both Western populations and the developing world. It was reported in a recent large series study that, in a young patient group of 46 patients, the average age at presentation was around 23 years (ranging from 4 to 45 years), with all patients presenting with lymphadenopathy and about 11% patients also showing extranodal disease [6]. In another 7-case series of EBV+ DLBCL involving Iraqi children, 6 children exhibited nodal diseases and 1 presented with extranodal bone involvement, with all children having advanced clinical stages [5]. In a 95-case series of DLBCL in Iran, 11.6% were found to be EBV-positive, with 7.5% and 14.5% occurring in young and old age groups, respectively; and there were no significant differences in immunohistochemical findings and clinical presentations between the young and old patient groups [7]. Thus, the 2016 revision of the WHO classification of lymphoid neoplasms has introduced the entity “EBV+ DLBCL, NOS,” replacing previous provisional entity “EBV-positive DLBCL of the elderly,” in recognition that such lymphomas may also occur in younger patients in addition to elderly adults [8].

EBV+ DLBCL in the young patients may be T-cell/histiocyte-rich large B-cell lymphoma-like, which is the most common in one series, gray-zone lymphoma-like with features intermediate between DLBCL and nodular sclerosis classical Hodgkin's lymphoma, and DLBCL, NOS [6]. Although there are other EBV+ reactive lymphoid hyperplasias and polymorphic extranodal or nodal lymphoproliferations [9], the architecture of EBV+ DLBCL is almost always diffuse with large cell proliferation, mixed with bystanding T-cells and histiocytes, with geographic necrosis and occasionally angiotropism [6, 10]. The EBV+ neoplastic B-cell tumors express B-cell associated antigens such as CD19, CD20, CD22, CD23, and CD79a and are often positive for CD30 and PD-L1 [6]. They usually exhibit nongerminal center or an activated B-cell phenotype negative for CD10 and positive for IRF4/MUM1 [5, 6]. The malignant large B-cells rather than the background reactive lymphocytes should express EBV [6, 10]. They are variably positive for EBER, LMP1, EBV latent membrane protein 2 (LMP2), EBV nuclear antigen 1 (EBNA1), and EBNA2 and are commonly associated with type II or type III EBV latency patterns [4]. Latency type II with positive LMP1 and negative EBNA2 expression is usually seen in immunocompetent patients. On the other hand, latency type III has unrestricted expression of all 6 EBV nuclear antigens, LMP1 and LMP2, and mainly occurs in immunosuppressed individuals, such as posttransplant or HIV-positive patients.

Our case is a very young child with an immature immune system. It may be the first reported EBV+ DLBCL occurring in a patient below 2 years of age. Her initial lymphoma process was primarily pulmonary. Even though lymphomatoid granulomatosis is also in the differential diagnosis with angiocentric and angiodestructive EBV+ lymphoma involving extranodal sites, it tends to have polymorphous lymphoid infiltrates and more prominent blood vessel wall invasion and occurs in adults and immunodeficient children. Her initial pulmonary EBV+ DLBCL further spread to her central nervous system without confirmed nodal involvement. Most of the lymphoproliferative disorders in immunocompetent children occur in nodal or nodal/extranodal sites. Instead of being in commonly observed nongerminal center phenotype, the patient had a germinal center type DLBCL. Although a full panel of EBV-related proteins and nuclear antigens were not analyzed, her lymphoma cells were positive for LMP1 and EBNA2. Thus, it is likely that the patient's EBV status was in latency type III, which usually indicates impaired immune functions and portends aggressive clinical behaviors [4, 6]. Although the patient did not have a well-defined history of immunodeficiency or genetic disorder, her lack of 12-month-old vaccinations made her prone to EBV infection, which appears to have been an acute primary infection at initial presentation. Her hypergammaglobulinemia, elevated IgG subclasses, decreased circulating lymphocyte subsets, and reduced total lymphocyte and CD3+ T-cell proliferative responses to PHA stimulation all support her having dysfunctional immune conditions. It is possible that EBV infection may have weakened her immature immune system and caused deficient immunoglobulin and cellular responses. The majority of young EBV+ DLBCL patients have a good prognosis, with more than 80% in clinical remission following chemotherapy [6]. About 8% of such young patients are reported to have died of the disease [6]. The presence of EBV is considered as an adverse prognostic factor compared to EBV-negative large B-cell lymphoma, along with CD30 immunopositivity in the tumor cells [11]. In addition to conventional R-CHOP (rituximab, cyclophosphamide, doxorubicin, vincristine, and prednisone) chemotherapy, with or without local radiation therapy [6], antiviral therapy, pathway specific therapy, and EBV-specific adoptive cellular immunotherapy are currently under investigation and hold promising future directions for treatment regimens [11].
